# Epigenetic Modulation, Intratumoral Microbiome, and Immunity in Early-Onset Colorectal Cancer

**DOI:** 10.1158/2767-9764.CRC-25-0177

**Published:** 2025-11-12

**Authors:** Ning Jin, Rebecca Hoyd, Ayse S. Yilmaz, Jiangjiang Zhu, Yunzhou Liu, Malvenderjit S. Jagjit Singh, Dennis J. Grencewicz, Xiaokui Mo, Matthew F. Kalady, Daniel W. Rosenberg, Caroline E. Dravillas, Eric A. Singer, John D. Carpten, Carlos H.F. Chan, Michelle L. Churchman, Nicholas Denko, Frances Di Clemente, Rebecca D. Dodd, Islam Eljilany, Naomi Fei, Sheetal Hardikar, Alexandra P. Ikeguchi, Anjun Ma, Qin Ma, Martin D. McCarter, Afaf E.G. Osman, Gregory Riedlinger, Lary A. Robinson, Bryan P. Schneider, Ahmad A. Tarhini, Gabriel Tinoco, Jane C. Figueiredo, Yousef Zakharia, Cornelia M. Ulrich, Aik Choon Tan, Daniel Spakowicz

**Affiliations:** 1 Division of Medical Oncology, Department of Internal Medicine, The Ohio State University Comprehensive Cancer Center, Columbus, Ohio.; 2 Department of Biomedical Informatics, The Ohio State University, Columbus, Ohio.; 3 Human Nutrition Program, Department of Human Sciences, The Ohio State University, Columbus, Ohio.; 4 Division of Colon and Rectal Surgery, The Ohio State University, Columbus, Ohio.; 5 Department of Medicine, UConn Health, Farmington, Connecticut.; 6 Division of Urologic Oncology, The Ohio State University Comprehensive Cancer Center, Columbus, Ohio.; 7 Department of Translational Genomics, University of Southern California, Los Angeles, California.; 8 Department of Surgery, Holden Comprehensive Cancer Center, The University of Iowa, Iowa City, Iowa.; 9 Aster Insights, Hudson, Florida.; 10 Department of Radiation Oncology, The Ohio State University Comprehensive Cancer Center, Columbus, Ohio.; 11 Department of Precision Medicine Oncology, Rutgers Cancer Institute of New Jersey, New Brunswick, New Jersey.; 12 Department of Internal Medicine, The University of Iowa, Iowa City, Iowa.; 13 Clinical Science Lab – Cutaneous Oncology, H. Lee Moffitt Cancer Center and Research Institute, Tampa, Florida.; 14 Department of Population Health Sciences, University of Utah Huntsman Cancer Institute, Salt Lake City, Utah.; 15 Department of Hematology/Oncology, Stephenson Cancer Center – The University of Oklahoma, Oklahoma City, Oklahoma.; 16 Department of Surgery, University of Colorado School of Medicine, Aurora, Colorado.; 17 Division of Hematology and Hematologic Malignancies, Department of Internal Medicine, University of Utah, Salt Lake City, Utah.; 18 Thoracic Oncology, H. Lee Moffitt Cancer Center and Research Institute, Tampa, Florida.; 19 Division of Hematology/Oncology, Indiana University Simon Comprehensive Cancer Center, Indianapolis, Indiana.; 20 Department of Cutaneous Oncology, H. Lee Moffitt Cancer Center and Research Institute, Tampa, Florida.; 21 Department of Immunology, H. Lee Moffitt Cancer Center and Research Institute, Tampa, Florida.; 22 Samuel Oschin Comprehensive Cancer Institute at Cedars-Sinai Medical Center, West Hollywood, California.; 23 Department of Oncological Sciences, University of Utah Huntsman Cancer Institute, Salt Lake City, Utah.; 24 Department of Biomedical Informatics, University of Utah Huntsman Cancer Institute, Salt Lake City, Utah.

## Abstract

The incidence of colorectal cancer in young adults (age of diagnosis <50 years) has been rapidly increasing. Although ∼20% of early-onset colorectal cancer (EOCRC) cases are due to germline mutations, the etiology of the majority of EOCRC cases remains poorly understood. Nongenetic factors such as environmental exposure and lifestyle changes are likely to have a direct link to the increased incidence of sporadic EOCRC. We hypothesize that such factors may be observable as alterations in the epigenome, microbiome, and immunome. We characterized the DNA methylation (DNAm) signature and measured DNAm age in EOCRC by using The Cancer Genome Atlas (TCGA). Furthermore, we carefully identified intratumoral microbes from TCGA and the Oncology Research Information Exchange Network datasets and then related the microbes to deconvolved immune cell abundances in EOCRC. We observed that the DNAm age in the EOCRC cohort was 12 years older when compared with the average-onset colorectal cancer (AOCRC) cohort, using three different epigenetic clocks. Differentially methylated sites associated with gene expression include cAMP-responsive element binding protein signaling in neurons, G protein–coupled receptor signaling, phagosome formation, and S100 family signaling. These differences were validated in the gene expression data from TCGA and the Oncology Research Information Exchange Network. When comparing the intratumoral microbes between EOCRC and AOCRC, no consistent differences were observed. Interestingly, the most abundant microbes interacted with the immune systems differently between the EOCRC and AOCRC tumors, characterized by more and larger positive correlations in EOCRC. These data suggest that epigenetic modulation and accelerated aging may play a key role in the development of EOCRC.

**Significance:**

We investigated whether environmentally driven factors contribute to EOCRC. We observed accelerated epigenetic aging in EOCRC and epigenetic changes associated with chronic inflammation. Tumor immune cell abundances correlated more strongly with microbes in EOCRC than in AOCRC. These data suggest a dysregulation of the immune response in EOCRC, driving chronic inflammation and tissue aging.

## Introduction

Colorectal cancer is the third most common cause of cancer deaths in both men and women in the United States. Although colorectal cancer incidence and mortality have decreased steadily among patients older than 65 years, the incidence of colorectal cancer in younger adults (<50 years old) has been rapidly increasing since the early 1990s (1). Approximately 20% of early-onset colorectal cancer (EOCRC) cases are attributed to germline DNA mutations that are related to familial cancer syndromes (2–4). The surge in colorectal cancer incidence in young adults is particularly alarming as the overall colorectal cancer frequency has decreased. There is no consensus on whether EOCRC is characterized by distinct molecular features compared with average-onset colorectal cancer (AOCRC), defined as patients with a colorectal cancer diagnosis at 50 years old or later. EOCRC is characterized by the advanced stage at diagnosis, poor cell differentiation, higher prevalence of signet ring cell histology, and distal location of the primary tumor (especially the rectum; refs. 5, 6). Available data suggest that survival is worse in patients younger than 30 years old, and sporadic colorectal cancer among the youngest patients represents a significant clinical challenge (7).

The etiology of sporadic EOCRC is poorly understood. Epidemiology studies estimate that approximately 50% to 60% of colorectal cancer cases in the United States are attributable to modifiable risk factors, such as smoking, alcohol, obesity, physical inactivity, and a Western diet (8–12). In one study, African Americans were found to have a higher colorectal cancer risk compared with South Africans living in rural areas (13). The higher colorectal cancer rates in African Americans were associated with diets higher in animal protein and fat and lower in fiber consumption (13). Diet also has a major impact on the composition and function of gut microbiota, which decompose and ferment dietary fibers to produce microbial metabolites, such as short-chain fatty acids, polyphenols, and vitamins. Changes in the gut microbiome composition (including bacteria, viruses, and fungi) may lead to various diseases, including irritable bowel syndrome, Crohn disease, and colorectal cancer (14–18). A diet high in fat and low in fiber has been associated with the disruption of intestinal bacterial homeostasis, which is termed intestinal dysbiosis (8, 12, 19). Studies have found that intestinal microbiome composition is more diverse in healthy individuals than in patients with colorectal cancer. Additionally, specific microbes have been linked to different stages of colorectal neoplasia, from adenomatous polyps to early-stage cancer to metastatic disease (20–25). For example, colorectal cancer-associated taxa such as *Fusobacterium* (anaerobic gram-negative bacteria) are frequently found in colorectal cancer tumors compared with adjacent normal colon tissue (26). Animal studies have shown that *Fusobacterium* can activate β-catenin signaling, promoting oncogenesis (27). Other pathogenic microbes, such as enterotoxigenic *Bacteroides fragilis*, can cause Th17-driven inflammation, leading to colonic tumor development (28). Therefore, changes in the intestinal microbiome and microbial metabolites may facilitate environmental risk factors that initiate and promote colorectal cancer (29, 30).

Numerous studies have found that microbial metabolites play important roles in modulating the host immune system (31–34). Intestinal microbiota are essential in developing and regulating innate and adaptive immunity (35, 36). Local immunity is promoted by recognizing pathogen-associated molecular patterns through pattern-recognition receptors in intestinal epithelial cells and innate immune cells within the gut. Bacterial and microbial metabolites can activate dendritic cells, which migrate to the draining lymph nodes to activate naïve T cells to form either T regulatory or Th17 cells (37). To maintain intestinal homeostasis, the immune system must tolerate antigens derived from commensal microbiota. Tolerance is achieved in part via the actions of T regulatory cells, which produce the anti-inflammatory cytokine IL10. In the setting of acute infection due to opportunistic pathogenic bacteria, the delicate balance of the commensal bacteria is disrupted (38), leading to the breakdown of the mucosal barrier and the translocation of gut bacteria to local lymph nodes and the peripheral circulation. Consequently, Th17 cells produce the proinflammatory cytokine IL17 and recruit neutrophils from the bloodstream, generating a profound inflammatory state (39). The chronic inflammatory state leads to the activation of key prosurvival and pro-proliferative signaling pathways via NF-κB, resulting in aberrant proliferation, epithelial cell transformation, and ultimately the development of colorectal cancer (40, 41). Therefore, the unique immune response to specific or pathogenic microbes may contribute to the development of EOCRC.

In addition to promoting colorectal cancer, microbial metabolites may influence the host epigenome, either by regulating the activity of epigenetic enzymes or by altering the abundance of cofactors needed for epigenetic modifications (42, 43). For instance, butyrate, one of the short-chain fatty acids produced by microbes, may inhibit histone deacetylases and colonic cell proliferation (44). Folic acid and other B vitamins from green leafy vegetables are the essential methyl donors required to provide substrates for DNA methylation (DNAm). Decreased dietary folate intake may deplete the levels of S-adenosyl methionine, resulting in DNA hypomethylation, which may lead to the activation of proto-oncogenes and chromosomal instability (42, 43). DNAm regulates gene transcription by either gene activation or silencing through DNA methyltransferases, which convert cytosine to 5-methylcytosine (45). DNAm at CpG islands in promoters is generally a repressive marker that prevents gene transcriptional activity by impeding the recruitment of transcriptional machinery. On the other hand, DNAm within intronic regions may cause increased transcription in a tissue-specific manner (46). Hypermethylation of specific regions, such as the CpG islands of tumor suppressor genes, plays an important role in carcinogenesis for many types of cancers, including colorectal cancer (43, 47–49). Studies have suggested that methylation changes may be the earliest alterations in the polyp-to-cancer transformation, suggesting that methylation may play a key role in colorectal cancer tumorigenesis (50–53). In fact, a study comparing 118 EOCRC cases with 225 AOCRC cases found hypomethylation in the long interspersed nuclear element-1 (a repetitive genomic element) in EOCRC (54). However, few studies have investigated the genome-wide epigenetic alterations that may underlie EOCRC.

In this study, our goal was to obtain further insight into the mechanisms associated with EOCRC. We hypothesized that EOCRC may be reflected by alterations in the intratumoral microbiome and DNAm, which are markers for environmental changes. We aimed to characterize the methylation signatures and investigate the unique distribution of the intratumoral microbiome and interactions between the microbiome and tumor-infiltrating lymphocytes in EOCRC.

## Materials and Methods

### The Cancer Genome Atlas dataset, methylation array, and epigenetic clock analysis

A total of 358 colorectal cancer cases, including 54 cases of EOCRC (age at diagnosis <50 years) and 304 cases of AOCRC (age at diagnosis ≥50 years), with matched methylation array [Infinium HumanMethylation450 (HM450)], RNA sequencing (RNA-seq; Illumina HiSeq) data from colon adenocarcinomas and rectal adenocarcinomas, and clinicopathologic information for each patient, were extracted from The Cancer Genome Atlas (TCGA).

DNAm profiles were downloaded from the Illumina Infinium HM450 platform using the TCGAbiolinks Bioconductor package (RRID:SCR_017683). DNAm values are reported as beta values for each CpG probe in each sample using Illumina BeadStudio software. Beta values are continuous variables between 0 and 1, representing the ratio of the intensity in which higher beta values suggest a higher level of DNAm (hypermethylation) and lower beta values suggest a lower level of DNAm (hypomethylation). All the methylation values are batch corrected using the ComBat function from the sva package (RRID:SCR_002155). Then, the difference in mean methylation between EOCRC and AOCRC groups for each probe is identified using the TCGAanalyzeDMR function in the TCGAbiolinks package (RRID:SCR_017683). The *P* value is calculated using the Wilcoxon test and adjusted using the Benjamini–Hochberg method. We performed a differential methylated CpG analysis to estimate the difference in DNAm for the probes between groups and their significance value using the Wilcoxon test and the Benjamini–Hochberg adjustment method. The differentially methylated sites are identified using a difference in methylation value |Δ beta| > 0.1 and a FDR-adjusted *P* value of < 0.05. We applied the batch effect removal tool ComBat to the level 3 methylation beta values (RRID:SCR_010974).

### Oncology Research Information Exchange Network dataset

The Oncology Research Information Exchange Network (ORIEN) is an alliance of 18 cancer centers in the United States established in 2014. All ORIEN alliance members utilize a standard Total Cancer Care (TCC) protocol. As part of the TCC study, participants provide written informed consent to have their clinical data followed over time, undergo germline and tumor sequencing, and be contacted by their provider if an appropriate clinical trial or other study becomes available. TCC is a prospective cohort study with a subset of patients enrolled in the ORIEN Avatar program, including research use only–grade whole-exome tumor sequencing, RNA-seq, germline sequencing, and deep longitudinal clinical data collection with lifetime follow-up. Studies are conducted in accordance with the Belmont Report. Nationally, more than 325,000 participants have enrolled in TCC. Aster Insights, ORIEN’s commercial and operational partner, harmonizes all abstracted clinical data elements and molecular sequencing files into a standardized, structured format to enable the aggregation of deidentified data for sharing across the network. The Ohio State University Institutional Review Board approved the study protocol (2015H0088), which is registered at ClinicalTrials.gov (NCT02482610).

ORIEN Avatar specimens undergo nucleic acid extraction and sequencing at HudsonAlpha or Fulgent Genetics. For frozen tissue DNA extraction, QIAGEN QIAsymphony DNA purification is performed, generating a 213-bp average insert size. For frozen and optimal cutting temperature tissue RNA extraction, the QIAGEN RNeasy Plus Mini Kit is used, generating a 216-bp average insert size. For formalin-fixed, paraffin-embedded tissue, a Covaris Ultrasonication FFPE DNA/RNA kit is utilized to extract DNA and RNA, generating a 165-bp average insert size. RNA-seq is performed using the Illumina TruSeq RNA Exome with single library hybridization, cDNA synthesis, library preparation, and sequencing (100 bp paired reads at HudsonAlpha, 150 bp paired reads at Fulgent Genetics) to a coverage of 100 million total reads/50 million paired reads.

Our study included 453 ORIEN Avatar patients with colon or rectal cancer who consented to the TCC protocol from the participating member sites of ORIEN. We filtered out all cases with microsatellite instability status in both TCGA and ORIEN datasets.

### Data processing for RNA sequencing

RNA-seq tumor pipeline analysis is processed according to the following workflow using the GRCh38/hg38 human genome reference sequencing and GENCODE build version 32. Adapter sequences are trimmed from the raw tumor sequencing FASTQ file. Adapter trimming via k-mer matching is performed along with quality trimming and filtering, contaminant filtering, sequence masking, guanosine/cytosine filtering, length filtering, and entropy filtering. The trimmed FASTQ file is used as input to the read alignment process. The tumor adapter–trimmed FASTQ file is aligned to the human genome reference (GRCh38/hg38) and the GENCODE genome annotation version 32 using the STAR aligner. The STAR aligner generates multiple output files for gene fusion prediction and gene expression (GE) analysis. RNA expression values are calculated and reported using estimated mapped reads, fragments per kilobase of transcript per million mapped reads, and transcripts per million mapped reads (TPM) at both the transcript and gene levels based on transcriptome alignment generated by STAR. The RSEM pipeline and GEs were quantified as TPM. GEs were log_2_ (TPM + 1) transformed, and downstream analyses were performed using the GE matrix.

For GE analysis, primary alignment is performed against the human genome reference GRCh38. GE values were quantified using the featureCounts tool of the Subread package version 1.5.1 (RRID:SCR_009803) in unstranded mode for genes described by the GENCODE annotation. Differential expression analysis was performed using limma/voom (RRID:SCR_010943). The functional analyses were generated using QIAGEN ingenuity pathway analysis (IPA; QIAGEN Inc., https://digitalinsights.qiagen.com/IPA; ref. 55). Cutoff values for the core analysis in IPA include *P* value < 0.05 and |Fold change| > 1.5. Gene count tables were deconvolved to immune cell abundances using CIBERSORTx (RRID:SCR_016955). Reference cell type signatures of individual lymphocytes were aggregated to estimate tumor-infiltrating lymphocytes.

### Microbe alignment and quantification with {exotic}

We utilized the tool {exotic} (56), which takes raw RNA-seq data (in this case, from the ORIEN and TCGA datasets) and carefully aligns it to both human and nonhuman reference genomes to identify low-abundance microbes. Models of association were analyzed based on each of the subgroups as well as all of the samples (the “all” group). We performed Cox proportional hazards regression to identify the microbes associated with overall survival. We evaluated 453 RNA-seq samples from ORIEN and then validated those associations on an independent dataset of 358 samples from TCGA, as previously described (56).

## Results

### Characteristics of patients with EOCRC from two datasets

We identified the methylation patterns using the TCGA dataset and correlated differentially methylated sites with GEs using the TCGA and ORIEN datasets. Then, we characterized intratumoral microbiome signatures of EOCRC from both TCGA and ORIEN datasets. We define EOCRC as age at diagnosis <50 and AOCRC as age at diagnosis ≥50. We collected an Infinium HM450 methylation array with matched RNA-seq data from TCGA, comprising a total of 358 colorectal cancer cases, including 54 EOCRC and 304 AOCRC cases (Table 1).

**Table 1.** Patient characteristics with colorectal cancer tumors in TCGA, stratified by the age of onset.

​	EOCRC	AOCRC	*P*
*N*	54	303	​
Sex = male (%)	28 (51.9)	167 (55.1)	0.768
Race (%)	​	​	0.246
American Indian or Alaska Native	0 (0.0)	1 (0.3)	​
Asian	3 (5.6)	8 (2.6)	​
Black or African American	11 (20.4)	40 (13.2)	​
Not reported	1 (1.9)	23 (7.6)	​
White	39 (72.2)	231 (76.2)	​
Primary diagnosis site (%)	​	​	0.454
Colon	38 (70.4)	227 (74.9)	​
Connective or soft tissues	0 (0.0)	2 (0.7)	​
Rectosigmoid junction	10 (18.5)	34 (11.2)	​
Rectum	6 (11.1)	40 (13.2)	​
Tumor location (%)	​	​	0.343
Left	30 (55.6)	140 (46.2)	​
Other	8 (14.8)	42 (13.9)	​
Right	16 (29.6)	121 (39.9)	​
Tumor stage (%)	​	​	0.046
Stage I	6 (11.5)	46 (16.1)	​
Stage II	13 (25.0)	114 (39.9)	​
Stage III	20 (38.5)	88 (30.8)	​
Stage IV	13 (25.0)	38 (13.3)	​
Histology (%)	​	​	0.238
Adenocarcinoma with mixed subtypes	1 (1.9)	1 (0.3)	​
Adenocarcinoma, NOS	42 (77.8)	256 (84.5)	​
Mucinous adenocarcinoma	10 (18.5)	32 (10.6)	​
Papillary adenocarcinoma, NOS	0 (0.0)	2 (0.7)	​
Tubular adenocarcinoma	1 (1.9)	12 (4.0)	​

Abbreviation: NOS, not otherwise specified.

We also obtained 453 colorectal cancer cases with RNA-seq from ORIEN, including 120 EOCRC and 333 AOCRC cases (Table 2). In both TCGA and ORIEN datasets, patients with EOCRC differed from patients with AOCRC by cancer stage, with patients with EOCRC having a more advanced stage at initial diagnosis (*P* value = 0.046 from TCGA; *P* value = 0.018 from ORIEN). In the ORIEN dataset, patients with EOCRC were found to have more left-sided primary tumor locations than right-sided tumor locations (*P* value = 0.008). Histologic subtypes, race, and sex were not significantly different between patients with EOCRC and AOCRC. Our observation of EOCRC presenting with advanced cancer stage and left-sidedness in our datasets is consistent with other research groups (5, 6).

**Table 2.** Patient characteristics with colorectal cancer tumors in ORIEN, stratified by the age of onset.

​	EOCRC	AOCRC	*P*
*N*	120	333	​
Sex = male (%)	64 (53.3)	184 (55.3)	0.798
Race (%)	​	​	0.601
American Indian or Alaska Native	0 (0.0)	3 (0.9)	​
Asian	5 (4.2)	15 (4.5)	​
Black or African American	11 (9.2)	19 (5.7)	​
Caucasian	99 (82.5)	282 (84.7)	​
Other	5 (4.2)	14 (4.2)	​
Primary diagnosis site (%)	​	​	0.568
Colon	48 (40.0)	140 (42.2)	​
Rectosigmoid colon	46 (38.3)	110 (33.1)	​
Rectum	26 (21.7)	82 (24.7)	​
Tumor location (%)	​	​	0.008
Left	85 (70.8)	218 (65.5)	​
Other	12 (10.0)	14 (4.2)	​
Right	23 (19.2)	101 (30.3)	​
Tumor stage (%)	​	​	0.018
Missing	15 (12.5)	42 (12.6)	​
Stage I	4 (3.3)	28 (8.4)	​
Stage II	18 (15.0)	85 (25.5)	​
Stage III	42 (35.0)	99 (29.7)	​
Stage IV	41 (34.2)	79 (23.7)	​
Histology (%)	​	​	0.889
Adenocarcinoma with mixed subtypes	0 (0.0)	1 (0.3)	​
Adenocarcinoma, NOS	111 (92.5)	299 (90.1)	​
Carcinoma	1 (0.8)	3 (0.9)	​
Mucinous adenocarcinoma	8 (6.7)	28 (8.4)	​
Papillary adenocarcinoma	0 (0.0)	1 (0.3)	​

Abbreviation: NOS, not otherwise specified.

### Epigenetic signatures and accelerated aging in EOCRC

**Figure 1.**
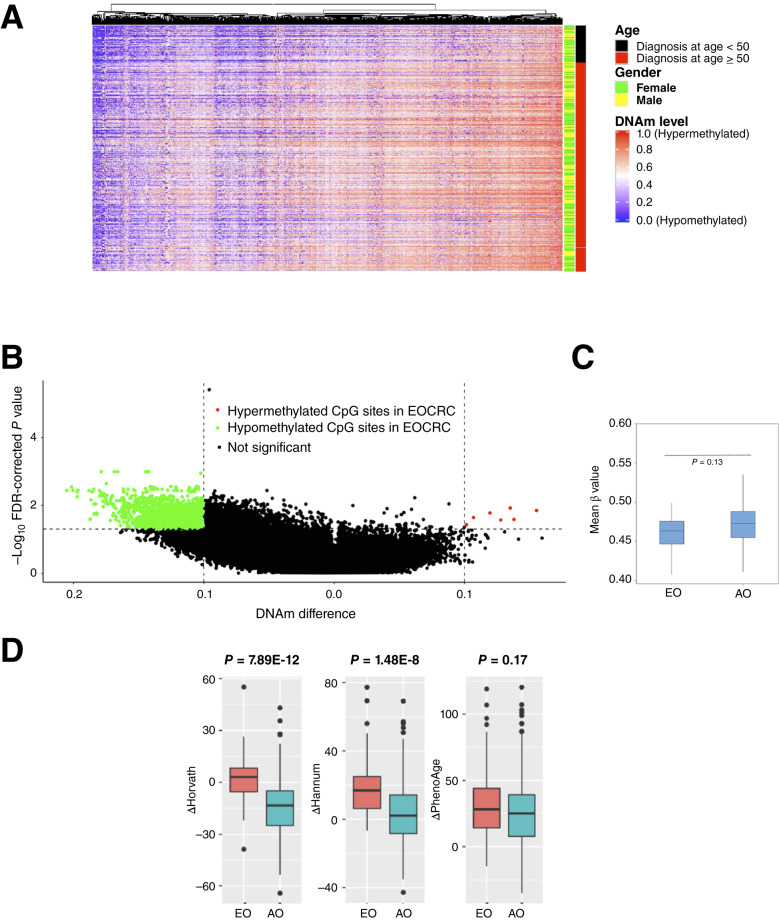
Accelerated aging with epigenetic signatures in EOCRC. **A,** Heatmap was drawn with samples (patients) in the row and CpG sites (probes) in the column. The DNAm values range from 0.0 (completely unmethylated, blue) to 1.0 (completely methylated, red). **B,** The volcano plot demonstrated differential methylated sites (expressed as β values) between EOCRC and AOCRC groups. △β for each CpG site is defined as β value of (EOCRC – AOCRC). FDR-adjusted *P* < 0.05 and |△β| ≥ 0.1 were used as cutoff values. Sites with △β value ≥0.1 and *P* value < 0.05 were defined as significantly hypermethylated sites, which were shown in red; those with adjusted *P* value < 0.05 and △β value ≤ −0.1 were defined as significantly hypomethylated sites (green). **C,** Box plot showed the mean β value of patients with EOCRC and AOCRC was 0.46 ± 0.028 and 0.47 ± 0.027 (*P* value = 0.13, two-sample *t* test), respectively. **D,** Box plots demonstrated the accelerated aging in EOCRC. The DNAm age acceleration (Δ = methylation age − chronologic age) in EOCRC and AOCRC for all samples is shown. The Δ_EOCRC_ predicted by Horvath, Hannum, and PhenoAge models was 2.1, 18.4, and 32.8 years, respectively. The Δ_AOCRC_ predicted by Horvath, Hannum, and PhenoAge was −14.2, 3.4, and 27.1 years, respectively, with *P* values of 7.89E−12, 1.48E−08, and 0.17, respectively.

We analyzed differentially methylated sites in the EOCRC and AOCRC groups using an Infinium HM450 methylation array in TCGA. The DNAm heatmap demonstrated the methylation patterns in EOCRC and AOCRC with the individual cases (patients; Fig. 1A). The volcano plot indicated more hypomethylated sites in the EOCRC group (Fig. 1B, per site differences and *P* values in Supplementary Table S1). However, the mean global methylation levels were not significantly different between the two groups (patients with EOCRC and AOCRC were 0.46 ± 0.028 and 0.47 ± 0.027, *P* value = 0.13, two-sample *t* test; Fig. 1C).

Prior studies showed that DNAm changes through the aging process (57–59). To minimize the confounding effect of methylation status solely due to age, we utilized epigenetic clocks to predict the methylation ages between patients with EOCRC and AOCRC. Epigenetic clocks are mathematical models used to estimate DNAm age, which can predict chronologic ages. We utilized three different epigenetic models, including Horvath (60), Hannum (61), and PhenoAge (62), to evaluate epigenetic aging in the EOCRC and AOCRC groups. We calculated DNAm age acceleration (Δ) as the difference between methylation age and chronologic age for each patient (Δ = methylation age − chronologic age). The DNAm age acceleration (Δ_EOCRC_) by Horvath, Hannum, and PhenoAge was 2.1, 18.4, and 32.8 years, respectively, with the average of Δ_EOCRC_ being 17.8 years. In comparison, the Δ_AOCRC_ calculated by Horvath, Hannum, and PhenoAge was −14.2, 3.4, and 27.1 years, respectively, with the average of Δ_AOCRC_ being 5.4 years. The difference between the average Δ_EOCRC_ and the average Δ_AOCRC_ is 12.4 years.

In other words, both EOCRC and AOCRC groups demonstrated age acceleration (with the exception of AOCRC by Horvath), and the methylation ages from EOCRC were more distant and older than their chronologic ages (*P* values of 7.89 × 10^−12^, 1.48 × 10^−8^, and 0.17, by Horvath, Hannum, and PhenoAge, respectively; Fig. 1D).

### Validation of the differential methylation as affecting gene expression

**Figure 2.**
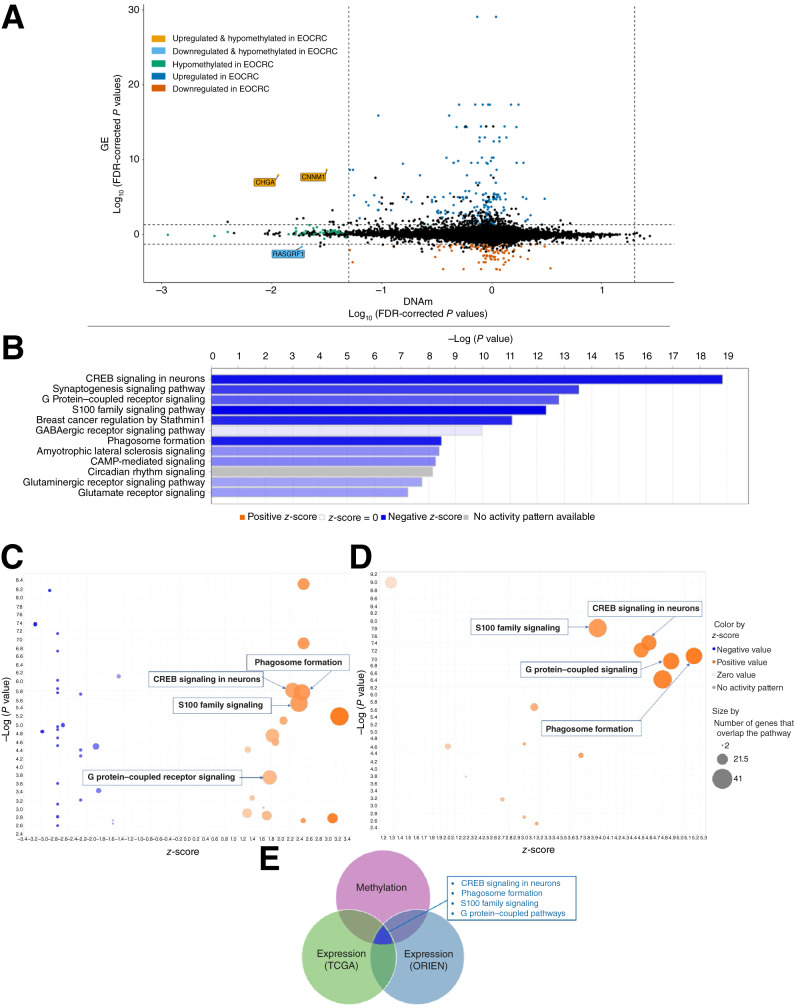
Methylation signature validation through GE profiles in EOCRC. **A,** Integration of the differentially methylated sites with paired RNA-seq showed genes that vary in methylation and transcription. The threshold for the FDR-adjusted *P* values for differences is 0.05 (FDR *P* value ≤ 0.05). Highlighted genes in orange indicate significant hypomethylation and expression. **B,** The canonical pathways involved with DMGs in EOCRC have an FDR-adjusted *P* value < 0.05. **C** and **D,** Bubble chart of pathways based on DEGs in EOCRC from TCGA and ORIEN, highlighting those predicted by methylation alone. The color of the pathways is based on their *z*-score, and the size of the bubble is correlated with the number of genes from the dataset that overlap each pathway. **E,** Summary of the analyses to validate the differential methylation, with those supported by all three datasets indicated within the box.

Hypermethylated CpG sites within the promoter regions commonly lead to the silencing of gene transcription and *vice versa*. To determine whether the DNAm differences between EOCRC and AOCRC led to functional effects in the cell (transcription), we integrated differential DNAm and GE using the starburst plot, which identifies genes in which methylation and expression levels are highly anticorrelated. The starburst plot shows nine distinct quadrants, in which the *x*-axis shows the FDR-adjusted *P* values for DNAm and the *y*-axis shows the FDR-adjusted *P* values for GE. The genes highlighted in the upper left and lower right quadrants show possible gene activation or silencing due to DNA hypomethylation or hypermethylation, with a difference in methylation >0.1 beta value and a difference in expression with fold change >2 between EOCRC and AOCRC (Fig. 2A, differential GE listed in Supplementary Table S2). Among the hypomethylated and upregulated genes in the EOCRC group, cyclin M1 and chromogranin A were identified as the key genes in which action may be due to DNA hypomethylation. Cyclin M1 is associated with neuron cell stemness and self-renewal, and the chromogranin A gene product modulates the neuroendocrine system.

To shed light on accelerated epigenetic aging in EOCRC, it is essential to investigate the biological pathways related to differential methylation in the EOCRC group. A total of 937 hypomethylated genes and 12 hypermethylated genes in EOCRC were identified; we utilized IPA to characterize pathways associated with methylation changes in EOCRC. The top five pathways include cAMP-responsive element binding protein (CREB) signaling in neurons, synaptogenesis signaling pathways, G protein–coupled receptor (GPCR) signaling, the S100 family signaling pathway, and breast cancer regulation by Stathmin1 (Fig. 2B). These pathways are involved with differential hypomethylated sites in EOCRC (Supplementary Table S3 for the list of differentially methylated CpG sites).

Next, we asked whether the pathways involved with differentially methylated genes (DMG) may modulate cellular transcription. We hypothesized that the pathways identified by the methylation analysis would be important if they also seemed significant from an unsupervised EOCRC versus AOCRC transcriptional analysis. Four of the pathways found to be important by methylation were found among the most differentially expressed gene (DEG) transcription in the TCGA (Fig. 2C) and ORIEN (Fig. 2D) datasets: CREB signaling in neurons, GPCR signaling, phagosome formation, and S100 family signaling pathways, as shown in the Venn diagram (Fig. 2E; Supplementary Tables S4 and S5 for GE in TCGA and ORIEN datasets, respectively). These pathways involved with DEGs were shown to be activated in IPA, which suggests that EOCRC-specific methylation patterns may strongly affect cellular transcription. As a note, the GE profiling used the unadjusted *P* value. The pathway analysis findings are exploratory and will be confirmed through our future experimental validation.

### Intratumoral microbes are not strongly associated with EOCRC

**Figure 3.**
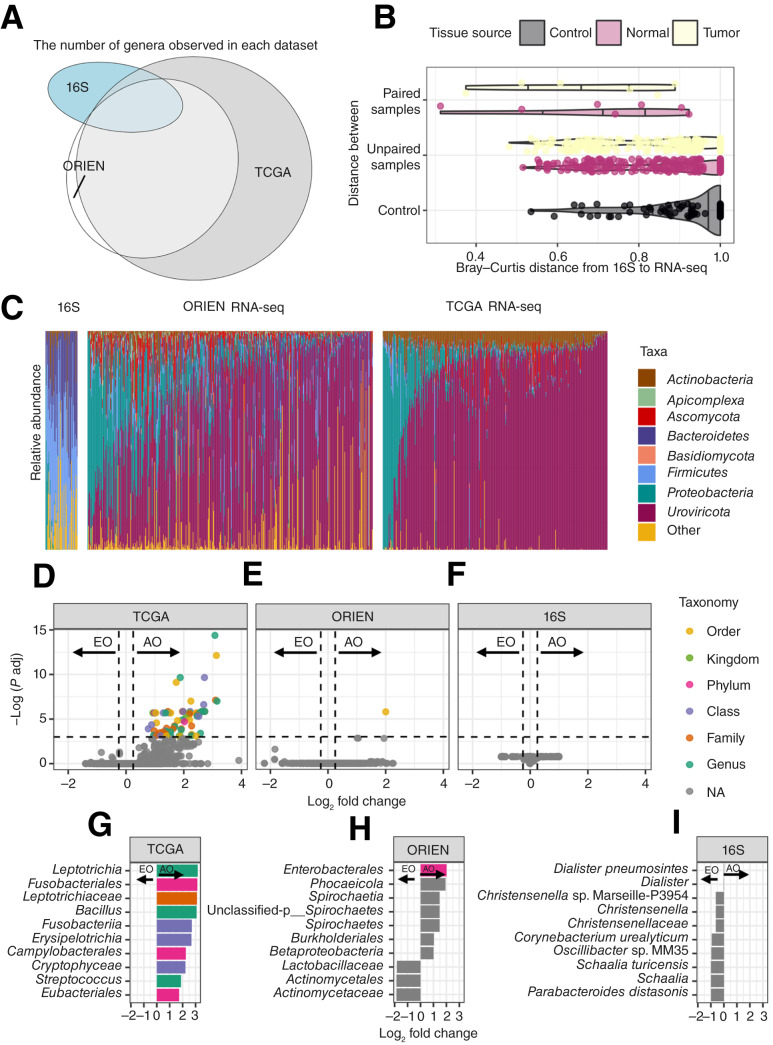
Intratumoral microbes do not show consistent differences in EOCRC. **A,** Venn diagram demonstrated partial overlap in microbes among three datasets. The abundances of observed genera that passed stringent quality and contamination filters are shown (ORIEN, *n* = 353 genera; TCGA, *n* = 357 genera; and 16S, *n* = 30 genera), with the best agreement between the two RNA-seq datasets. **B,** The Bray–Curtis dissimilarity plot showed that RNA-seq–derived microbes were most similar to 16S microbes from the same tumor (paired) and least similar to microbes from adjacent normal tissue from another person (unpaired). **C,** The comparison of the relative abundance of microbes between the 16S-based dataset and the RNA-seq–based datasets (ORIEN and TCGA). **D–F,** Volcano plots showed no consistent differences in microbes from RNA-seq–based (TCGA, ORIEN) or 16S-based datasets. No microbes passed the significance threshold in EOCRC. **G–I,** Effect sizes of the most significant taxa across the three analyses showed no similarities in the AOCRC-enriched microbes.

Compared with AOCRC, patients with EOCRC have unique gene signatures of phagosome formation and S100 family signaling pathways based on canonical pathways associated with DMGs and DEGs. The S100 family proteins are expressed in different cell types (immune and epithelial cells) and regulate cellular processes, including proliferation, inflammation, and invasion (63–65). We next sought to determine if the epigenetic aging effects, which seemed to be driven by inflammatory expression programs, could be attributed to another environmentally derived factor: the microbiome. We used the {exotic} (56) pipeline to count nonhuman reads in bulk RNA-seq data from TCGA and ORIEN. To validate the presence of microbes and provide an additional assessment of the EOCRC versus AOCRC tumor microbe burden, we generated 16S amplicon data from 60 samples that partially overlap with the ORIEN dataset. The microbes identified in each dataset overlapped, with the most similarity between the RNA-seq datasets (Fig. 3A). Roughly half of the microbes identified in the 16S data were not observed in the RNA-seq results, which may be due to the lower depth of coverage expected in bulk RNA-seq data (i.e., greater sensitivity in the 16S data) or, conversely, the 16S data observing microbes that are not transcriptionally active. As an additional validation, we compared the distance between the microbial compositions of samples from the same patient (paired, Fig. 3B) and different patients (unpaired). The RNA-seq–derived microbes were more similar to 16S from the same tumor than to other colorectal cancer samples (Kruskal–Wallis rank-sum test *P* value = 0.02508). Furthermore, the RNA-seq–derived microbes were not significantly different from those derived from 16S rRNA sequencing of tumors or adjacent normal tissue (Kruskal–Wallis rank-sum test, *P* value = 0.6682). In the normal samples, the paired samples are more similar than the unpaired samples (*P* = 0.03181). All groups differed significantly from negative controls (all *P* values < 0.01). This gave us the confidence to analyze the RNA-seq data further for EOCRC and AOCRC differences.

*Firmicutes* dominated the tumor microbes in the 16S-derived group, and the 16S samples showed a more diverse set of phyla (i.e., more “Other”), likely due to the increased sensitivity of bacterial communities (Fig. 3C). In the RNA-seq–derived groups, there were large differences in the number of viruses detected (not detectable by 16S). *Uroviricota* and *Proteobacteria* are predominant microbes in both ORIEN and TCGA datasets. *Actinobacteria* are enriched only in TCGA but not in ORIEN. *Uroviricota* is a phylum of viruses, specifically a group of tailed bacteriophages, that is a major component of the human gut virome and has been shown to influence the composition and function of the bacterial community (66). *Proteobacteria*, *Fusobacteria*, and *Actinobacteria* can be found in colorectal cancer tissues, and their presence can be associated with cancer development, progression, and survival in patients with colorectal cancer (67, 68).

Finally, comparing the enrichment of EOCRC with AOCRC by abundance showed no microbes enriched in EOCRC across the three datasets [Fig. 3D–F, per microbe log_2_ fold changes and *P* values in Supplementary Tables S6 (TCGA and ORIEN) and S7 (16S)], including *Fusobacterium* abundance (Supplementary Fig. S1). Furthermore, the microbes enriched in AOCRC varied in each dataset (Fig. 3G–I), indicating no consistent relationship between AOCRC and a specific microbe. The community abundances did not significantly relate to the primary tumor location (e.g., colon, rectum) in either the TCGA or ORIEN datasets (Permutational Multivariate Analysis of Variance, *P* value 0.301 and 0.68, respectively; Supplementary Fig. S2A and S2B). Despite a larger sample size, only a single organism, *Enterobacterales*, was enriched in AOCRC for the ORIEN dataset (Fig. 3E and H). This may be due to the wider geographic distribution from which patient samples were collected relative to the TCGA or 16S datasets.

### Immune cell relationship with microbes in EOCRC

**Figure 4.**
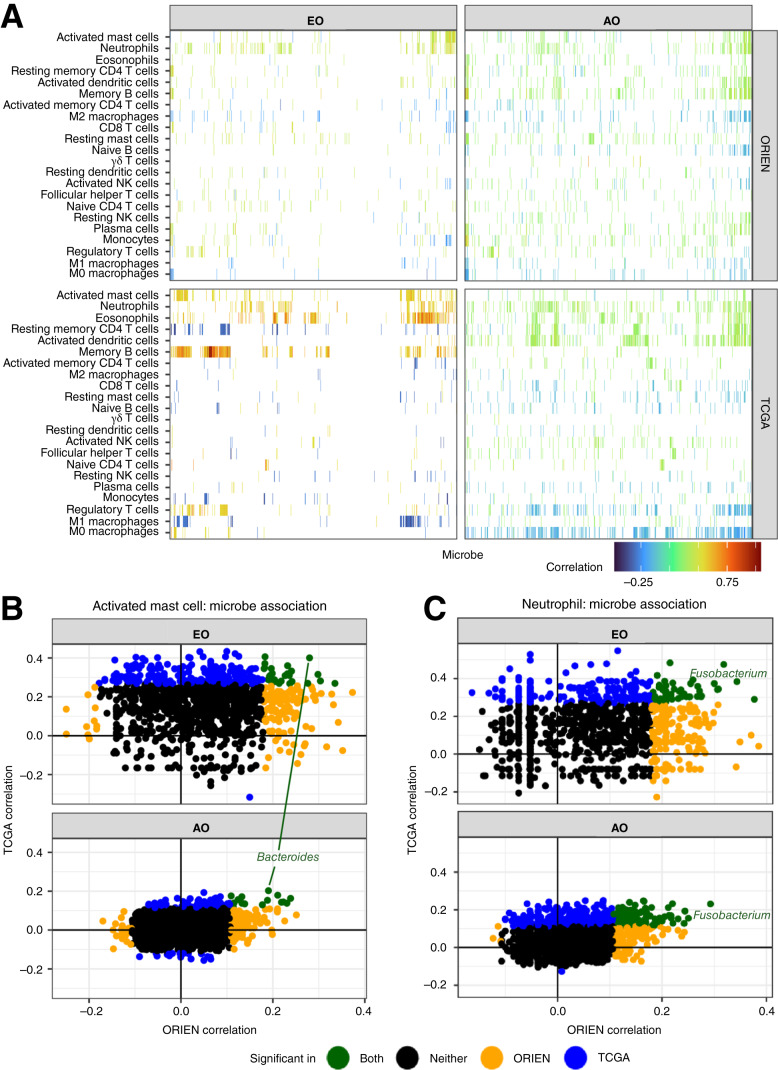
Relationship between microbes and immune cell composition. **A,** All microbe-genus level abundances (columns) correlated to the deconvolved immune cell abundances (rows), with the strength of the association and its direction indicated by color in the TCGA (top row) and ORIEN (bottom row) datasets. Spearman correlations between the deconvolved abundance of (**B**) activated mast cells or (**C**) neutrophils and microbes in both the TCGA and ORIEN datasets. Concordant associations (significant in both and in the same direction) are shown in green, with select taxa labeled. There were no microbes significantly anticorrelated with activated mast cells or neutrophils.

Despite the lack of EOCRC-specific microbe enrichment, the gene signatures in EOCRC led us to explore whether the inflammatory state observed in EOCRC might be due to interactions with microbes. To test this, we estimated the immune cell composition of all tumors by deconvolution and then correlated the immune cell abundances (Supplementary Tables S8 and S9) with the microbial abundances at the genus level. Few estimated immune cell abundances were statistically different between the EOCRC and AOCRC groups in the ORIEN dataset (activated dendritic cells, *P* value = 0.043; M1 macrophages, *P* value = 0.009, Supplementary Fig. S3), and none in the TCGA datasets (Supplementary Fig. S4). Surprisingly, the EOCRC tumors showed stronger correlations than AOCRC in both the ORIEN and TCGA datasets (Fig. 4A). These correlations were observed across many microbes—rather than just a few strong relationships—consistent with the cohort’s lack of specific microbe enrichment (Fig. 3D–H). Activated mast cells were the most strongly correlated with many EOCRC microbes consistently between the ORIEN and TCGA datasets (Fig. 4A). Specific microbes showed consistent correlations with some immune cells but with a larger scale in EOCRC. For example, *Fusobacterium* spp. was the most significantly correlated microbe with neutrophils in both the TCGA and ORIEN datasets, as well as in both EOCRC and AOCRC tumors; however, the correlation size in EOCRC was larger (Fig. 4B; Supplementary Table S10). Other associations were inconsistent between the two datasets, with eosinophils and memory B cells most strongly associated with microbes in the ORIEN dataset (Fig. 4A). At the same time, neutrophils were most strongly correlated in the TCGA dataset (Fig. 4C; Supplementary Table S10). This suggests that the EOCRC tumors respond to microbes differently and more robustly than AOCRC tumors in a way consistent with innate immune activation and chronic inflammation.

## Discussion

Epigenetic modifications, including histone modification and DNAm, can modulate gene transcription by altering chromatin structure and DNA accessibility without changing the DNA sequence. DNAm is not accurately maintained over cell divisions and leads to methylation changes (69). Early studies showed that global levels of methylation increase in the infant years and then decrease starting in late adulthood (70–72). With the development of microarray and second-generation sequencing technologies, later studies characterized the aging pattern as global methylation loss and focal gains in specific promoter-associated CpG islands (73–75). Based on this observation, mathematical models have been developed to utilize these aging-specific CpG sites to predict chronologic age and age-related health outcomes, including cardiovascular diseases, neurodegenerative diseases, cancers, and all-cause mortality.

As sporadic EOCRC is thought to be related to environmental influences and lifestyle changes, we sought to examine methylation alterations in EOCRC. We utilized epigenetic clocks to evaluate if there is a difference in epigenetic aging between EOCRC and AOCRC groups by mining the TCGA Infinium HM450 methylation array database. Our study showed accelerated aging in EOCRC cases compared with AOCRC cases. We found that DNAm age acceleration Δ_EOCRC_ (methylation age − chronologic age) computed by different epigenetic clock models was 12 years older than patients with Δ_AOCRC_. Certainly, there are variations in predicted methylation ages using different epigenetic models, and it will remain challenging to select the best model to evaluate accelerated aging in EOCRC.

Our result is consistent with the study findings from the Buchanan Group, in which they demonstrated accelerated aging in EOCRC using their unique methylation signature comprising 234 methylation loci (76). Our analysis demonstrated that the majority of DMGs are hypomethylated with the unique methylation signatures in EOCRC cases, including CREB signaling in neurons, synaptogenesis signaling pathways, GPCR signaling, phagosome formation, the S100 family signaling pathway, and breast cancer regulation by Stathmin1. Correspondingly, the canonical pathways involved with DEGs partially overlap with the DNAm profile in EOCRC, including CREB signaling in neurons, GPCR signaling, phagosome formation, and the S100 family signaling pathway. Pathways involved with DEGs were upregulated, corresponding to hypomethylated DMGs. These overlapping pathways between DNAm and GE indicate that epigenetic modulation (influenced by environmental risk factors) may lead to the development of EOCRC.

It is well known that epigenetic alterations are one of the hallmarks of aging (77), and neurodegenerative diseases may cause accelerated aging by inducing DNA damage, oxidative stress, inflammation, and metabolic dysregulation (78–81). CREB, a transcription factor, acts as a downstream effector of the GPCR–protein kinase A (PKA)–CREB signaling pathway. CREB has been shown to play an essential role in promoting cell proliferation, neuronal survival, and synaptic plasticity in the central nervous system (82–84), and dysregulated CREB signaling may be associated with cognitive deficits and aging (85). GPCR–PKA–CREB signaling has been shown to play a role in maintaining stemness and promoting progression and metastasis, and CREB knockdown reduces the metastatic potential in colorectal cancer (86, 87). However, it is unknown whether DNAm plays a role in regulating GPCR–PKA–CREB signaling in EOCRC and accelerated aging.

The S100 family proteins are involved in the cell cycle, cytoskeleton activity, cell differentiation and motility, inflammation, and/or antimicrobial activity (88). They are associated with neurologic disorders, cancers, and cardiac and inflammatory diseases (89). Several S100 proteins, including S100A1, S100A2, S100A4, and S100A11, were found in colon carcinomas with more aggressive disease and worse clinical outcomes (90, 91). S100A4 can activate NF-κB signaling and the Wnt–β-catenin pathway to promote tumorigenesis and metastasis in colorectal cancer (92, 93). Knockdown of the S100 protein by lentiviral RNA interference can inhibit colon cancer growth and metastasis (94). Earlier studies also demonstrated that DNAm modulated gene transcription regulation in S100 functions in different cancers, including colon cancer (95, 96). Supported by these abundant research findings, we plan to investigate the S100 family signaling pathway in EOCRC in future studies.

Gut microbiota play a central role in various host physiologic and metabolic functions, including fiber degradation and fermentation, energy supply, lipid metabolism, vitamin synthesis, and maintenance of intestinal barrier integrity (97–100). Intestinal dysbiosis can cause an increased inflammatory state, produce toxic metabolites, and promote an immunosuppressive tumor microenvironment that suppresses antitumor immune surveillance (101–105). Chronic inflammation activates key pro-proliferative signaling pathways via NF-κB, resulting in aberrant proliferation and epithelial cell transformation, thereby promoting carcinogenesis (40, 41). It has been established that certain pathogenic microbes, including *Escherichia coli*, *Enterococcus faecalis*, *B. fragilis*, and *Fusobacterium nucleatum*, are increased in patients with colorectal cancer (68). Therefore, the link between a Western diet and dysbiosis has stimulated considerable interest in uncovering alterations in microbial community structure in EOCRC. Several recent studies, however, failed to identify enrichment of *F. nucleatum* in the patient population with EOCRC, which is consistent with our study (106–108). Our results did not identify enrichment of specific microbes in EOCRC across both the TCGA and ORIEN datasets. Further studies are required to determine whether intestinal dysbiosis may have a direct association with EOCRC.

Interestingly, when comparing the microbes with inferred immune cell abundances, the EOCRC microbes showed more larger correlations with immune cells despite the same immune cell–microbe relationships being found in EOCRC and AOCRC (e.g., *Fusobacterium* was consistently associated with neutrophils in both EOCRC and AOCRC). This implies that EOCRC may respond more strongly to the presence of microbes, driving a proinflammatory phenotype, inflammaging, and earlier cancer development. Furthermore, this suggests an approach to increase immune tolerance to gut microbes that may protect against EOCRC.

Our study has several limitations. We utilized TCGA for methylation and TCGA and ORIEN for microbiome analysis as a discovery and validation approach. However, technical differences between the datasets may have led to a higher false negative rate than a single, larger dataset with uniform data generation procedures and processing. In addition, some ORIEN tumor samples (127 out of 453 samples, 28% of the total cases) were from metastatic sites, such as the liver, lung, pelvis, peritoneum, and ovary. These metastatic sites were most likely from tissue biopsies to confirm disease recurrence (stage IV). This will compromise the microbiome analysis due to the inconsistency of the intratumoral microbiome between primary colorectal cancer sites and metastatic sites even though some microbes from metastatic sites may inherit from the tissue of origin (109, 110). Another limitation is the lack of microbiome source material, likely from the gut and responding to diet and lifestyle factors. A thorough treatment would include paired collection of stool as well as mucosa-adherent and luminal microbiome samples. In addition, a set of normal colonic tissue samples from healthy donors or paired normal adjacent tissue would be ideal to serve as controls to evaluate cancer-associated microbes and epigenetic aging. Furthermore, the study remains strictly correlative. Our findings need further validation in large cohorts of patients, as well as mechanistic studies in animal models to identify causality.

### Conclusions

We found accelerated aging in EOCRC by mining the TCGA datasets. Methylation ages predicted by three different epigenetic clocks in patients with EOCRC were, on average, 12 years older than methylation ages in patients with AOCRC. Canonical pathways involved with differential methylation and GE partially overlapped, including CREB signaling in neurons, GPCR, phagosome formation, and S100 family signaling pathways in EOCRC. These four pathways were found to be hypomethylated and activated in gene transcription in both TCGA and ORIEN datasets. There was no significant intratumoral microbiome identified in EOCRC. However, specific microbes (such as *Fusobacterium*) showed consistent correlations with certain immune cells (neutrophils) on a larger scale in EOCRC. In summary, our findings suggested that DNAm changes and epigenetic aging may contribute to the development of EOCRC.

## Supplementary Material

Supplementary Table S1 LIST OF DIFFERENTIALLY METHYLATED SITES IN EOCRC VS AOCRC FROM TCGA

Supplementary Table S2 LIST OF GENES WITH HYPOMETHYLATION AND TRANSCRIPTION UPREGULATION IN STARBURST PLOT

Supplementary Table S3 LIST OF DIFFERERNTIALLY METHYLATED GENES GROUPED INTO CANNONICAL PATHWAYS IN EOCRC BY USING IPA

Supplementary Table S4 LIST OF GENES GROUPED INTO CANNONICAL PATHWAYS IN EOCRC FROM TCGA BY USING IPA

Supplementary Table S5 LIST OF GENES GROUPED INTO CANNONICAL PATHWAYS IN EOCRC FROM ORIEN BY USING IPA

Supplementary Table S6 LIST OF MICROBES IN TCGA AND ORIEN BY RNA-SEQ

Supplementary Table S7 LIST OF MICROBES BY 16S

Supplementary Table S8 CORRELATION BETWEEN IMMUNE CELLS AND MICROBES IN ORIEN

Supplementary Table S9 CORRELATION BETWEEN IMMUNE CELLS AND MICROBES IN TCGA

Supplementary Table S10 CORRELATION BETWEEN SUBSETS OF IMMUNE CELLS AND MICROBES IN ORIEN AND TCGA

Supplementary Figure S1 Supplementary Figure S1 showed Fusobacterium abundance by age status across the TCGA and ORIEN datasets.

Supplementary Figure S2 Supplementary Figure S2 showed microbial composition by tumor location.

Supplementary Figure S3 Supplementary figure S3 showed estimated immune cell composition in tumors stratified by EO vs AO CRC in the ORIEN dataset.

Supplementary Figure S4 Supplementary Figure S4 showed estimated immune cell composition in tumors stratified by EO vs AO CRC in the TCGA dataset.

## Data Availability

The Ohio State University Institutional Review Board approved data access in an Honest Broker protocol (2015H0185) and TCC protocol (2013H0199) in coordination with Aster Insights. The processed data generated in this study are publicly available using BioProject accession code PRJNA856973. Analysis scripts and data to regenerate all figures and tables are available at: https://github.com/spakowiczlab/exorien-exogieo. Other data generated in this study are available from the corresponding author upon request.
