# Supplementary material for: Epigenetic Modulation, Intratumoral Microbiome, and Immunity in Early-Onset Colorectal Cancer
Source: Cancer Res Commun. 2025 Nov 12;5(11):1985–97. doi: 10.1158/2767-9764.CRC-25-0177 (PMC12606411; doi:10.1158/2767-9764.CRC-25-0177)
Supplement: Supplementary Figure S1 — showed Fusobacterium abundance by age status across the TCGA and ORIEN datasets. [file crc-25-0177_supplementary_figure_s1_suppsf1.pptx]

## Slide 1
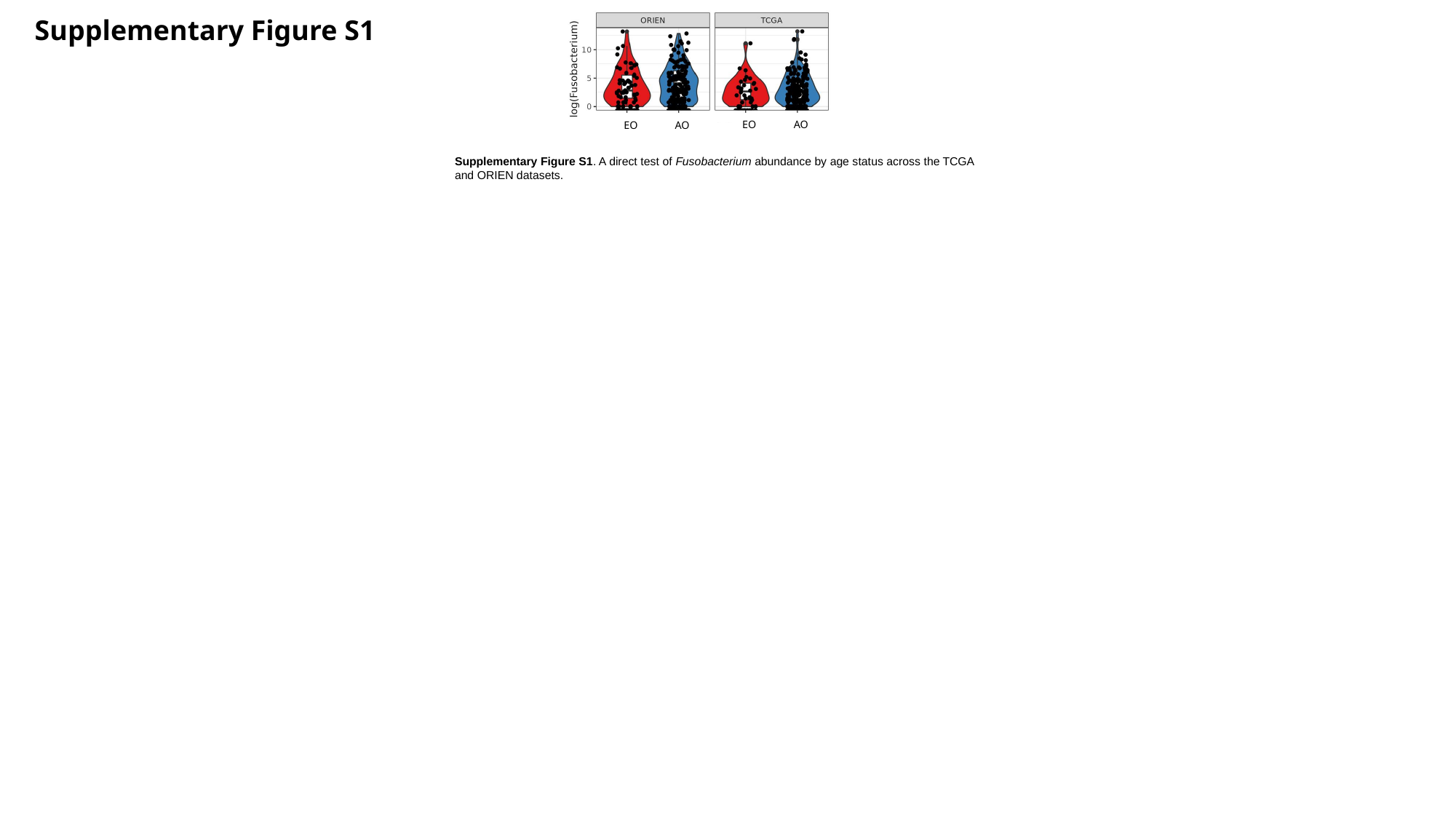

Supplementary Figure S1
AO
EO
AO
EO
Supplementary Figure S1. A direct test of Fusobacterium abundance by age status across the TCGA and ORIEN datasets.
