# Supplementary material for: Epigenetic Modulation, Intratumoral Microbiome, and Immunity in Early-Onset Colorectal Cancer
Source: Cancer Res Commun. 2025 Nov 12;5(11):1985–97. doi: 10.1158/2767-9764.CRC-25-0177 (PMC12606411; doi:10.1158/2767-9764.CRC-25-0177)
Supplement: Supplementary Figure S2 — showed microbial composition by tumor location. [file crc-25-0177_supplementary_figure_s2_suppsf2.pptx]

## Slide 1
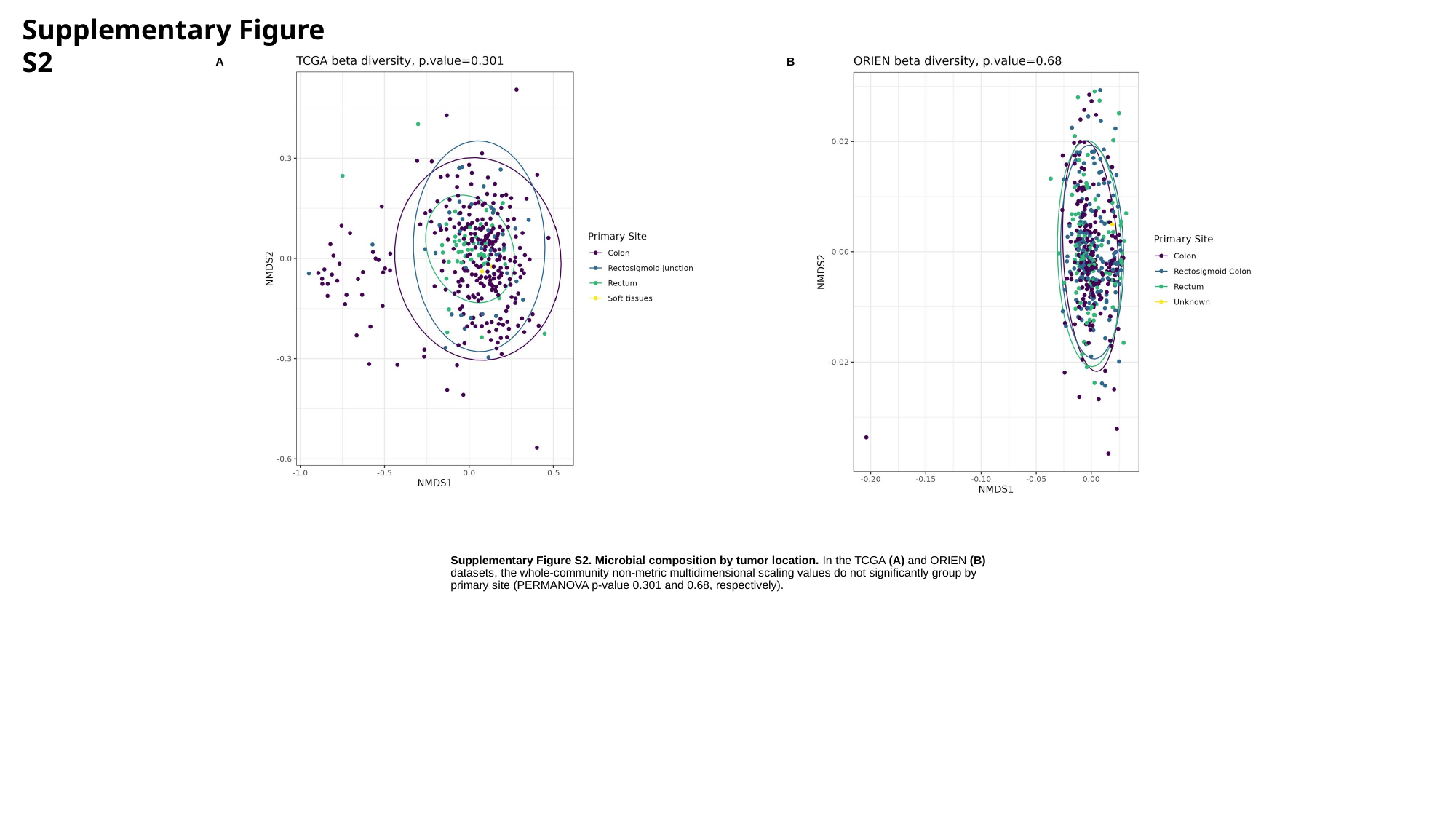

Supplementary Figure S2
B
A
Supplementary Figure S2. Microbial composition by tumor location. In the TCGA (A) and ORIEN (B) datasets, the whole-community non-metric multidimensional scaling values do not significantly group by primary site (PERMANOVA p-value 0.301 and 0.68, respectively).
