# Supplementary material for: Epigenetic Modulation, Intratumoral Microbiome, and Immunity in Early-Onset Colorectal Cancer
Source: Cancer Res Commun. 2025 Nov 12;5(11):1985–97. doi: 10.1158/2767-9764.CRC-25-0177 (PMC12606411; doi:10.1158/2767-9764.CRC-25-0177)
Supplement: Supplementary Figure S3 — showed estimated immune cell composition in tumors stratified by EO vs AO CRC in the ORIEN dataset. [file crc-25-0177_supplementary_figure_s3_suppsf3.pptx]

## Slide 1
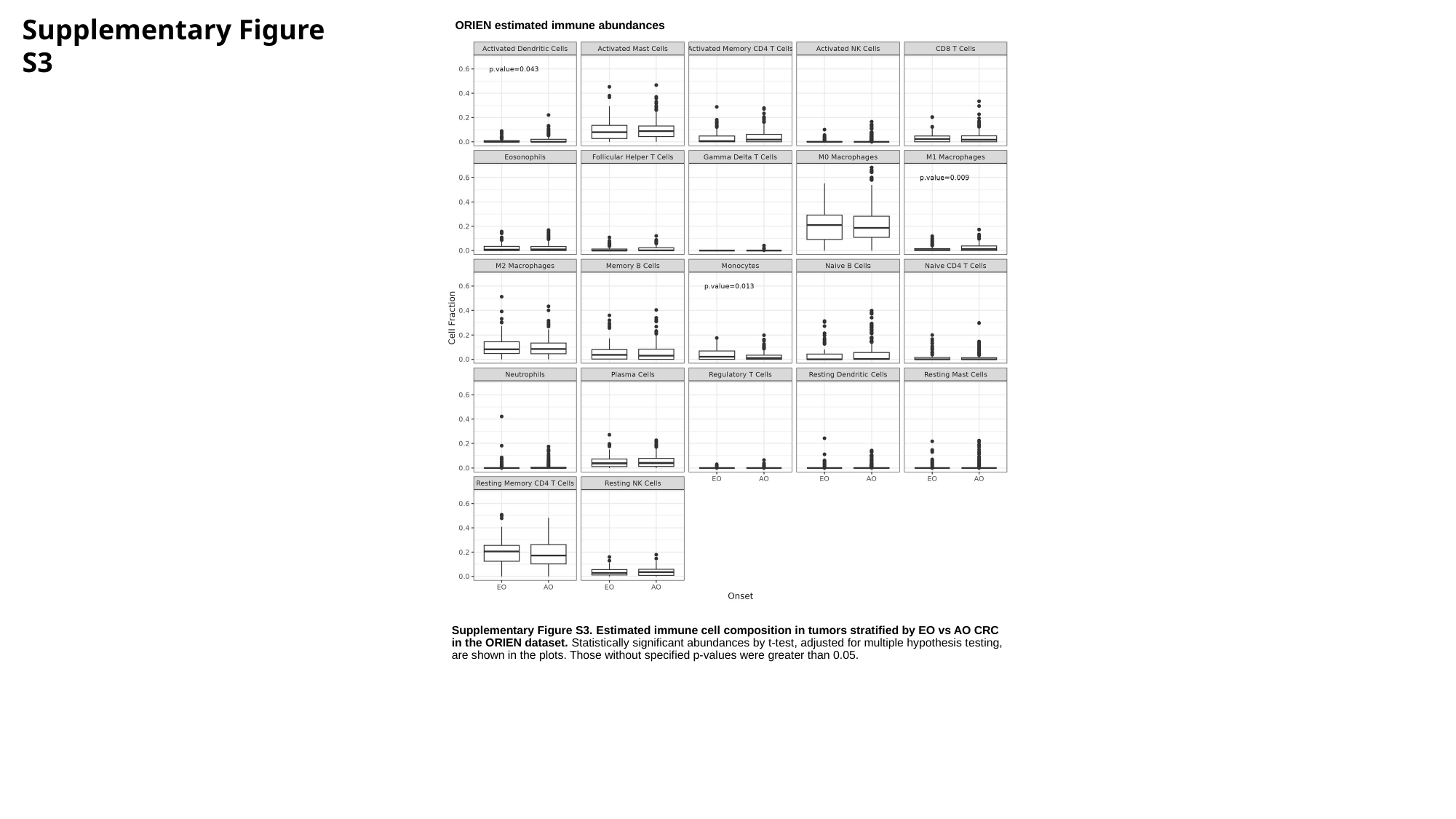

Supplementary Figure S3
# ORIEN estimated immune abundances
Supplementary Figure S3. Estimated immune cell composition in tumors stratified by EO vs AO CRC in the ORIEN dataset. Statistically significant abundances by t-test, adjusted for multiple hypothesis testing, are shown in the plots. Those without specified p-values were greater than 0.05.
