# Supplementary material for: Epigenetic Modulation, Intratumoral Microbiome, and Immunity in Early-Onset Colorectal Cancer
Source: Cancer Res Commun. 2025 Nov 12;5(11):1985–97. doi: 10.1158/2767-9764.CRC-25-0177 (PMC12606411; doi:10.1158/2767-9764.CRC-25-0177)
Supplement: Supplementary Figure S4 — showed estimated immune cell composition in tumors stratified by EO vs AO CRC in the TCGA dataset. [file crc-25-0177_supplementary_figure_s4_suppsf4.pptx]

## Slide 1
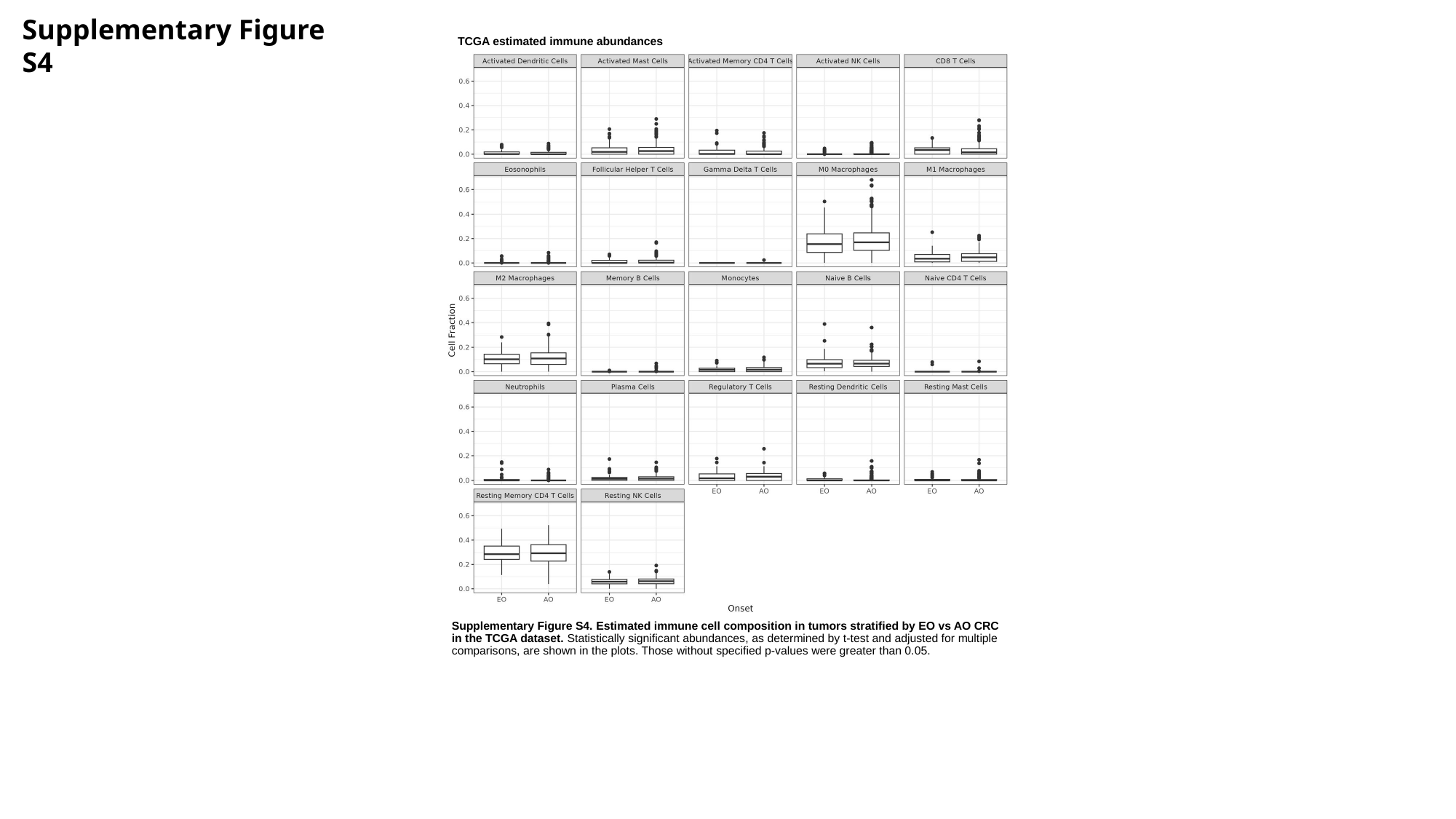

Supplementary Figure S4
TCGA estimated immune abundances
Supplementary Figure S4. Estimated immune cell composition in tumors stratified by EO vs AO CRC in the TCGA dataset. Statistically significant abundances, as determined by t-test and adjusted for multiple comparisons, are shown in the plots. Those without specified p-values were greater than 0.05.
